# The Einstellung effect in anagram problem solving: evidence from eye movements

**DOI:** 10.3389/fpsyg.2014.00679

**Published:** 2014-07-02

**Authors:** Jessica J. Ellis, Eyal M. Reingold

**Affiliations:** Department of Psychology, University of Toronto MississaugaMississauga, ON, Canada

**Keywords:** eye movements, problem solving, anagrams, Einstellung effect, insight problems

## Abstract

The Einstellung effect is the counterintuitive finding that prior experience or domain-specific knowledge can under some circumstances interfere with problem solving performance. This effect has been demonstrated in several domains of expertise including medicine and chess. In the present study we explored this effect in the context of a simplified anagram problem solving task. Participants solved anagram problems while their eye movements were monitored. Each problem consisted of six letters: a central three-letter string whose letters were part of the solution word, and three additional individual letters. Participants were informed that one of the individual letters was a distractor letter and were asked to find a five-letter solution word. In order to examine the impact of stimulus familiarity on problem solving performance and eye movements, the central letter string was presented either as a familiar three-letter word, or the letters were rearranged to form a three-letter nonword. Replicating the classic Einstellung effect, overall performance was better for nonword than word trials. However, participants’ eye movements revealed a more complex pattern of both interference and facilitation as a function of the familiarity of the central letter string. Specifically, word trials resulted in shorter viewing times on the central letter string and longer viewing times on the individual letters than nonword trials. These findings suggest that while participants were better able to encode and maintain the meaningful word stimuli in working memory, they found it more challenging to integrate the individual letters into the central letter string when it was presented as a word.

## INTRODUCTION

The concept that stimulus familiarity and previously acquired domain knowledge might impair problem solving performance has been referred to by a variety of interrelated terms including functional fixedness, negative transfer, mental set, and Einstellung. Functional fixedness refers to cases where familiarity with habitual uses of objects blocks other uses from being considered. For example, in the classic “candle-box” insight problem introduced by Duncker (1945) the presented use of the box as a container is hypothesized to interfere with the required consideration of the box as a shelf for supporting the candle. Similarly, negative transfer refers to the notion that the retrieval of previously acquired stimulus–response associations can impair the establishment and maintenance of new stimulus-response associations (e.g., Schultz, 1960; Sweller, 1980; Chrysikou and Weisberg, 2005; Landrum, 2005; Osman, 2008). Finally, a problem solving set or mental set refers to the negative impact of prior exposure to similar problems (either pre-experimental or during the experiment), which triggers a familiar but inappropriate solution and prevents alternative solutions from being considered. The Einstellung effect (*Einstellung* is German for attitude), which was originally demonstrated by Luchins’ (1942) seminal series of water jar experiments, constitutes an excellent illustration of the negative impact of a mental set (for a review see Bilalić and McLeod, 2014). In this paradigm, habitual approaches to problem solving are induced through exposure to multiple problems that have similar solution methods. When a problem is subsequently presented for which the habitual solution method is not appropriate, many participants claim that the problem is unsolvable. However, naive participants can find the solution quickly, thus showing that the problem is not intrinsically difficult and that the difficulty experienced by solvers reflects the negative impact of prior experience.

When considered in the context of human expertise, the idea that prior experience and stimulus familiarity might interfere with problem solving performance seems at first blush to be rather counterintuitive. This is because there is a large body of research demonstrating that stimulus familiarity and domain-specific knowledge acquired through extensive and deliberate practice underlie the superior performance of experts relative to their less skilled counterparts (for a review see Ericsson and Charness, 1994). However, experts are not immune to the negative impact of prior experience and stimulus familiarity as demonstrated in studies of expertise in medicine (de Graaff, 1989; Croskerry, 2003; Gordon and Franklin, 2003) and chess (Saariluoma, 1992; Reingold et al., 2001b; Bilalić et al., 2008a,b, 2010; Sheridan and Reingold, 2013; Bilalić and McLeod, 2014). For example, Bilalić et al. (2008a) employed eye movement monitoring to study the Einstellung effect in chess experts. Players were required to find a checkmate with the fewest number of moves. There were two possible solutions: a familiar five-move sequence and a less well-known three-move sequence (the optimal solution). After identifying the familiar solution, expert chess players reported that they were searching for the optimal one. However, the eye movement record revealed that their attention continued to be directed more often towards chess board regions involved in the familiar rather than the optimal solution. Thus, it appears that the Einstellung effect demonstrated in chess experts was due to the familiar scenario activating a schema in memory that directs attention towards information relevant to itself, and away from other information (Bilalić et al., 2008a, 2010; Bilalić and McLeod, 2014).

In the present study, in order to further investigate the negative influence of stimulus familiarity on problem solving, we monitored participants’ eye movements while they performed a modified anagram problem solving task that was introduced by Ellis et al. (2011). Anagram tasks provide a unique opportunity to study the Einstellung effect in a domain of expertise possessed by most adults, that is, their familiarity with words. In addition, unlike most problem solving tasks that were employed to study the Einstellung effect, the use of anagrams allows for the creation of a large number of independent trials in which an Einstellung effect might occur. Anagrams have long been used to study insight problem solving (for a review see Ellis et al., 2011) as well as to demonstrate the negative impact of a mental set on problem solving performance (e.g., Rees and Israel, 1935; Maltzman and Morrisett, 1952; Kaplan and Schoenfeld, 1966; Juola and Hergenhahn, 1967). In particular, it has been established (e.g., Beilin and Horn, 1962; Ekstrand and Dominowski, 1965, 1968; Tresselt and Mayzner, 1965; Mayzner and Tresselt, 1966) that solution rates are lower and response times are slower when the solution word (e.g., HEART) is scrambled to create a word anagram (e.g., EARTH) than a nonword anagram (e.g., THREA). It is likely that the familiar word anagram produces activation (orthographic, phonological, lexical, and/or semantic) that is irrelevant to the solution and hinders the decomposition and restructuring operations that are required to produce the solution word (e.g., Hollingworth, 1935; Devnich, 1937).

**FIGURE 1:**
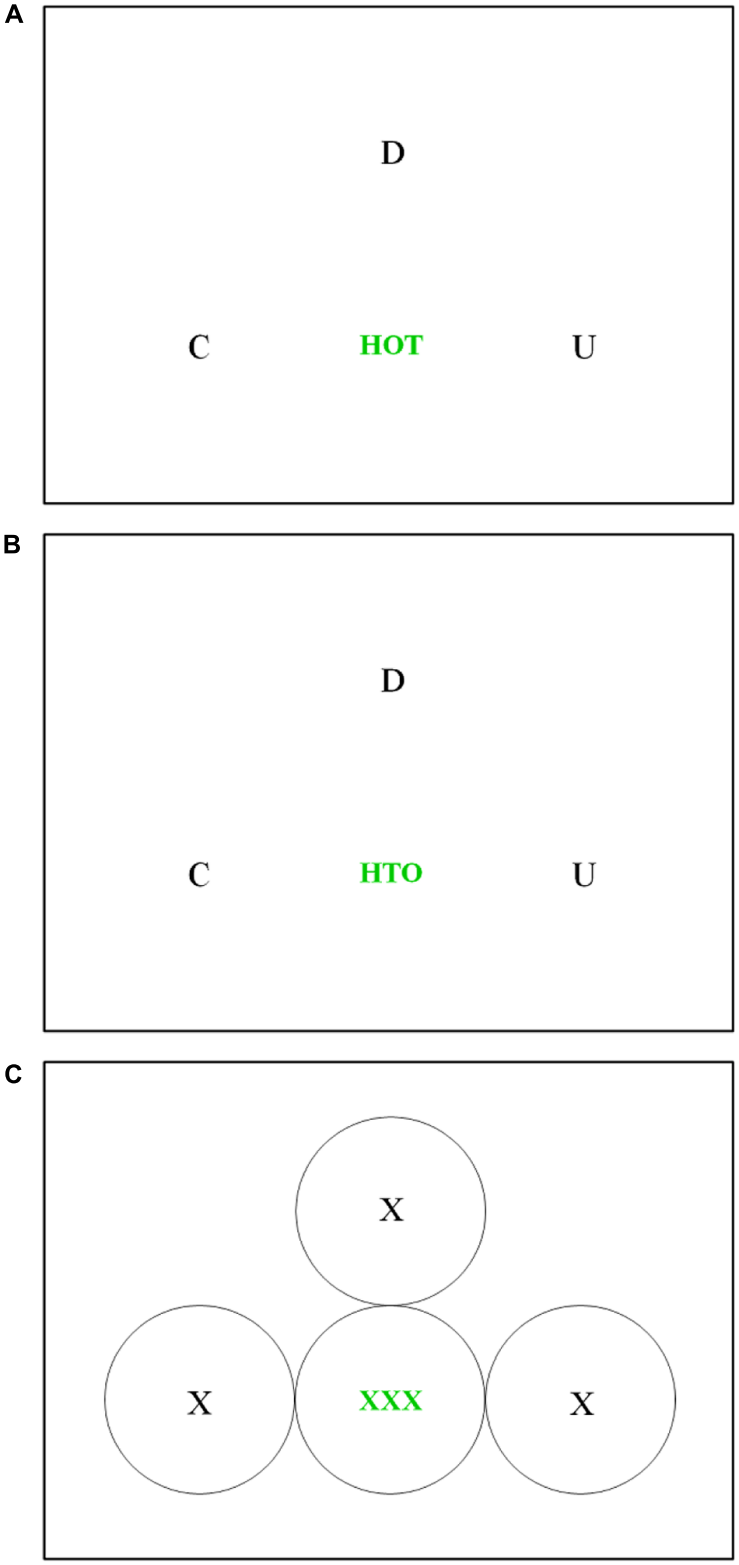
**Examples of the anagram problem stimulus display, shown with the central letter string arranged as a word (A) and as a meaningless letter string (B), and an illustration of the circular interest areas within which fixations were assigned to the individual letters or the central letter string, shown here as Xs (C).** The solution to the anagram problems is TOUCH.

The present investigation involved eye movement monitoring during anagram problem solving. As illustrated in **Figure 1**, the anagram task we used consisted of six uppercase letters: a centrally located three-letter string, plus three individual letters positioned above and to either side of the central letter string. The central letter string could be arranged either as a familiar three-letter word, or as a meaningless string of three letters. Participants were asked to produce a five-letter solution word, and were informed that one of the individual letters was a distractor letter that was not part of the solution word. Using a similar anagram task, Ellis et al. (2011) reported that near the beginning of trials, viewing times on the distractor and solution letters were indistinguishable, meaning that participants did not immediately perceive that the distractor letter was irrelevant to the solution. Towards the end of trials, viewing times on the distractor letter decreased relative to the solution letters, indicating that partial solution knowledge had developed, and this change occurred several seconds prior to solution. Importantly, the pattern of viewing times was the same regardless of whether or not participants reported a subjective experience of insight upon solution, thereby demonstrating a dissociation between the subjective experience of insight and the objective accumulation of solution knowledge. In the present study we expected to replicate the pattern reported by Ellis et al. (2011). In addition, based on previous findings with anagrams (e.g., Beilin and Horn, 1962; Ekstrand and Dominowski, 1965, 1968; Tresselt and Mayzner, 1965; Mayzner and Tresselt, 1966) we predicted better problem solving performance when the central letter string was presented as a nonword than a word. Finally, we explored differences in the pattern of looking behavior as a function of the familiarity of the central letter string.

## MATERIALS AND METHODS

### PARTICIPANTS

Sixty undergraduates from the University of Toronto Mississauga participated in exchange for partial course credit or $10. All participants had normal or corrected-to-normal vision and were fluent English speakers.

### APPARATUS

An SR Research EyeLink 1000 eye tracking system was used to record participants’ eye movements with a sampling rate of 1000 Hz. The stimuli were displayed on a 19-inch Viewsonic monitor with a refresh rate of 75 Hz and a screen resolution of 1024 × 768. Participants were seated 60 cm from the display and used a chinrest with a head support to minimize head movement. Following calibration, gaze-position error was less than 0.5°.

### MATERIALS

Anagram problems consisted of six uppercase letters: a centrally located three-letter string, plus three individual letters positioned above and to either side of the central letter string (see **Figure 1** for examples). All three letters in the central letter string belonged to the solution word, while only two of the individual letters belonged to the solution word, with the third being a randomly placed distractor letter. The task was to combine two of the three individual letters with the central letter string to create a five-letter solution word. Each anagram problem had only one possible solution, meaning that the distractor letter did not allow for the formation of any alternative five-letter words.

Each anagram problem could be presented in either “word” or “nonword” condition. In the “word” condition, the central letter string consisted of a three-letter word, while in the “nonword” condition, the central letter string consisted of a scrambled nonword version of those same three letters (see **Figure 1**). For each anagram problem, the identity and location of the three individual letters was the same across both conditions, such that the only difference between a given anagram problem in the two conditions was the configuration of the central letter string as a word or nonword. In the “word” condition, the central letter string words had a mean frequency of 435 per million (SD = 1243) according to Brysbaert and New (2009).

Solution words were made up of five unique letters, always began with a consonant, and contained either one vowel (33% of problems) or two vowels (67% of problems). Solution words had a mean frequency of 175 per million (SD = 396) according to Brysbaert and New (2009). The central letter string always consisted of two consonants and a vowel, as did the three individual letters. In order to remind participants that the three letters in the central letter string must always be included in the solution word, these letters were displayed in green, in a slightly smaller and bolder font than the three individual letters, which were displayed in black. Each anagram problem subtended approximately five visual degrees in height and 14 visual degrees in width.

The location of the individual distractor letter was counterbalanced across anagram problems. In an attempt to avoid any a priori bias away from the distractor letter, we matched the distractor letter with the other two individual solution letters in terms of letter frequency (averaged across all five possible letter positions within the solution word) using tables by Mayzner and Tresselt (1965). Across all experimental anagrams, the mean frequency of the distractor letter was no different from the mean frequency of the individual solution letters (distractor *M* = 193, SD = 91, solution *M* = 199, SD = 115, *t* < 1).

### PROCEDURE

Participants completed six practice trials followed by 72 experimental trials. Half the anagram problems were presented in the “word” condition and half were presented in the “nonword” condition, and both anagram order and central letter string type were randomized for each participant. Across participants, each anagram problem was presented an equal number of times in the “word” and “nonword” conditions.

Every trial began on a blank screen with a central fixation cross. After 1000 ms, the anagram problem appeared and remained on the screen until a response was made, or until the trial timed out after 45 s. Participants were instructed that speed of responding was of utmost importance and were discouraged from verifying their solution prior to response, even if that might elicit the occasional incorrect solution. Participants pressed a button on the response pad in order to respond. The stimulus display then disappeared and participants verbalized their answer to the experimenter, who provided feedback as to whether or not their response was correct.

After every trial, participants were asked to classify their subjective experience of solving the anagram problem. Participants selected one of the following options (from Novick and Sherman, 2003) by pressing a corresponding button on the response pad.

1 “The solution came to mind suddenly, seemingly out of nowhere. I have no awareness of having done anything to try to get the answer.”
2 “I tried various letter arrangements in order to solve the anagram, but none of them seemed to work. Then the solution came to mind suddenly.”
3 “I tried various letter arrangements in order to solve the anagram. I was able to build on one of these arrangements to work out the solution step by step.”
4 “I did not solve the anagram.”

We considered options 1 and 2 to describe subjective experiences of insight, and labeled all trials where participants selected option 1 or 2 as “popout” trials. Option 3 does not describe a subjective experience of insight, so trials where participants selected option 3 were labeled “non-popout” trials. Participants made another button press to advance to the next trial at their own pace.

## RESULTS

Our main focus in this experiment involved examining the effect of central letter string type (word vs. nonword) on mean task performance and eye movement measures. However, we also wanted to ensure that manipulating central letter string type did not alter the nature of the problem solving task as compared to prior findings (Ellis et al., 2011). Accordingly, while we primarily focus on the differences between word and nonword trials, we also examined any interaction between the familiarity of the central letter string and participants’ subjective experience of insight (i.e., trials in which the solution was experienced as emerging suddenly were classified as popout trials, whereas trials in which the solution was experienced as gradual were classified as non-popout trials; see Method section for details). Across participants, 52.7% of trials were correct, 4.2% of trials were incorrect, and 43.1% of trials timed out with no solution. Mean response time for correct trials was 14.3 s (SD = 3.3 s), with 69.0% of correct trials classified by participants as popout, and 31.0% classified as non-popout. The effect of central letter string type on overall problem solving performance is summarized in **Table 1**. Importantly, response times were significantly slower for word trials than for nonword trials. In addition, there was a numerical trend toward lower accuracy for word trials than nonword trials, although this difference did not reach significance. Finally, there was no impact of stimulus familiarity on the subjective experience of insight problem solving, as shown by the virtually identical proportion of word trials and nonword trials that were classified as popout.

**Table 1:** Mean problem solving and eye movement measures shown overall for correct trials, and separately for word and nonword trials.

Variable	Overall		Word	Nonword	Significance
Accuracy (% correct)	52.7 (2.4)		51.7 (2.4)	53.8 (2.6)	*t*(59) = 1.52, n.s.
Response time (s)	14.3 (0.4)		15.3 (0.6)	13.4 (0.4)	*t*(59) = 3.25, *p* < 0.01
Number of dwells, central	7.39 (0.37)		8.11 (0.41)	6.76 (0.37)	*t*(59) = 4.69, *p* < 0.001
Number of dwells, letters	9.40 (0.55)		10.45 (0.63)	8.53 (0.53)	*t*(59) = 4.80, *p* < 0.001
Percentage of trials classified as popout	69.0 (3.4)		68.9 (3.5)	69.2 (3.6)	*t* < 1, n.s.

*SEs are shown in parentheses.*

Eye movement analyses were performed only on correct trials, and only included fixations that could be assigned to a particular item in the stimulus display. Specifically, a fixation was assigned to an individual letter or to the central letter string if it fell within a 192 pixel diameter circle around that item (these fixation areas did not overlap; for an illustration, see **Figure 1C**). Within correct trials across participants, 69.9% of fixations were assigned to the central letter string, 28.3% of fixations were assigned to one of the three individual letters, and 1.8% of fixations could not be assigned to either the central letter string or the individual letters. Assigned fixations were then converted to dwells, where a dwell is defined as one or more consecutive fixations within the same area prior to an eye movement to another area. As shown in **Table 1**, corresponding to the slower response times for word than nonword trials, there was a greater number of dwells per trial on both the central letter string and the individual letters for word trials as compared to nonword trials, revealing the classic negative influence of familiarity on problem solving performance.

**FIGURE 2:**
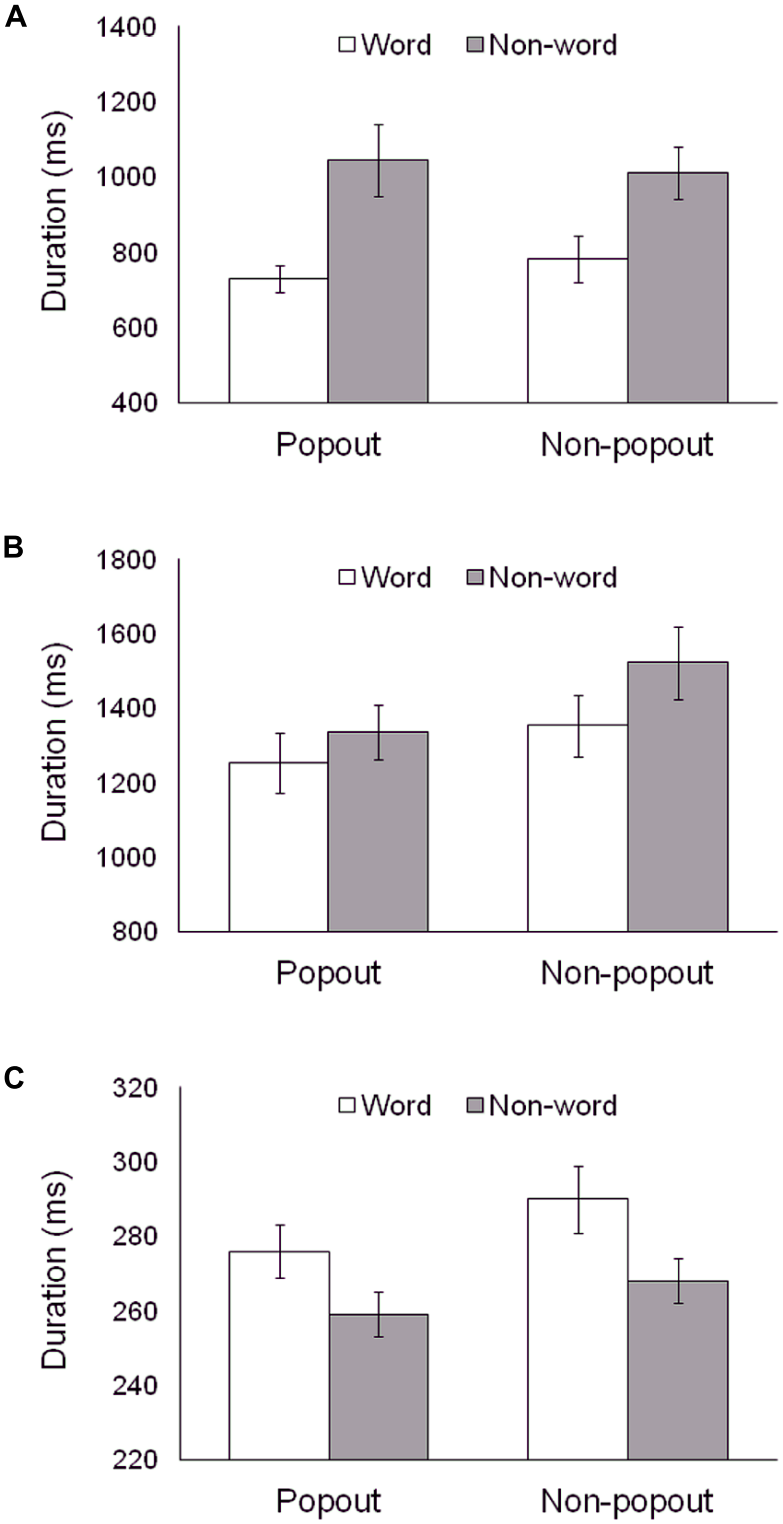
**Mean eye movement measures for word and nonword conditions within popout and non-popout trials: initial latency on the central letter string (A); subsequent dwell duration on the central letter string (B); and dwell duration on individual letters (C)**.

However, several fine-grained eye movement measures, shown in **Figure 2**, revealed a more complex pattern of the effects of central letter string type. More specifically, we calculated overall means for the following eye movement measures: (a) duration of the initial latency on the central letter string (i.e., the interval from stimulus onset until the first eye movement that exited the central letter string area); (b) dwell duration on the central letter string during subsequent revisits; and (c) dwell duration on the individual letters. For each eye movement measure, we carried out a 2 × 2 ANOVA with subjective report (popout vs. non-popout) and central letter string type (word vs. nonword) as independent variables. As can be seen in **Figure 2**, some eye movement measures revealed facilitation for word trials relative to nonword trials, while other eye movement measures revealed interference. In addition, there were no significant interactions between central letter string type and subjective report for any eye movement measure (all *F*s < 1.45, n.s.), indicating that the differences between word and nonword trials were the same for both popout and non-popout trials. Specifically, the initial latency on the central letter string was much shorter for word trials than for nonword trials (**Figure 2A**; *F*(1,59) = 29.78, *p* < 0.001), and this processing advantage for words over nonwords was also present for subsequent dwells on the central letter string (**Figure 2B**; *F*(1,59) = 7.78, *p* < 0.01).This processing advantage for word trials is likely due to a working memory advantage in encoding and maintaining the central letter string when it is arranged as a word as compared to a nonword. In marked contrast, dwell duration on individual letters revealed a processing disadvantage for word trials. Specifically, dwells on individual letters were longer for word trials than for nonword trials [**Figure 2C**; *F*(1,59) = 25.31, *p* < 0.001]. This processing disadvantage might be due to difficulty in integrating the individual letters into the central letter string when it is in the form of a unitary gestalt.

**FIGURE 3:**
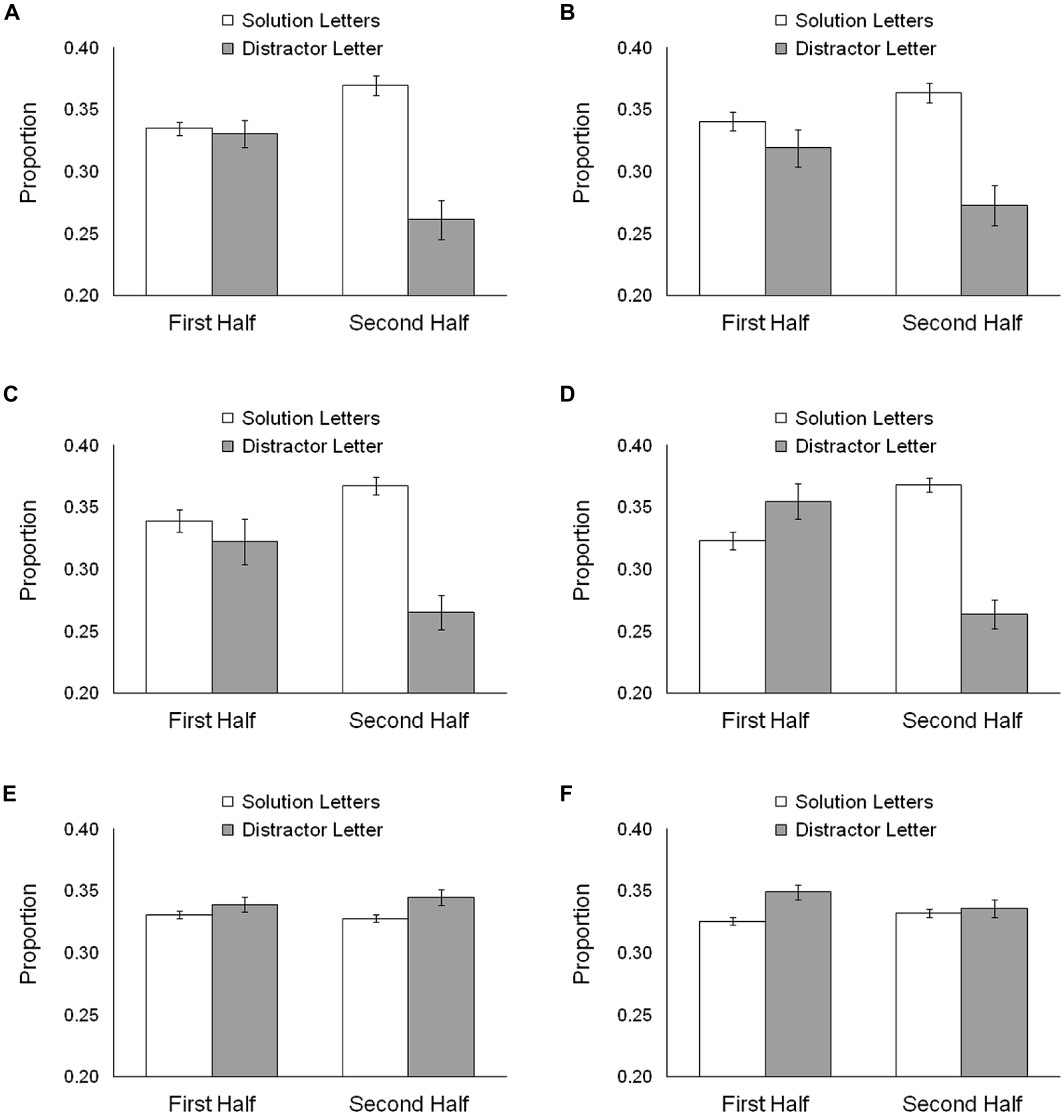
**Proportion of viewing time on the solution letters and the distractor letter in the first and second half of trials for each combination of central letter string type (word in A,C,E; nonword in B,D,F) and trial type (popout in A,B; non-popout in C,D; unsolved in E,F)**.

In addition, we contrasted viewing times on the distractor and solution letters during the first half and second half of trials as a function of both the familiarity of the central letter string (word vs. nonword) and the reported subjective experience (popout vs. non-popout). Based on the findings reported by Ellis et al. (2011), viewing times for the distractor letter and the solution letters were expected to be the same at the beginning of trials, whereas solution knowledge towards the end of trials should result in lower viewing times on the distractor letter as compared to the solution letters. To examine this prediction, we compared the proportion of time spent on the distractor letter and the mean of the two solution letters separately for the first and second half of trials. Accordingly, we conducted 2 × 2 × 2 ANOVAs on the proportion of viewing time with letter type (distractor vs. solution), subjective report (popout vs. non-popout) and central letter string type (word vs. nonword) as independent variables. As can be seen in **Figures 3A–D**, for all conditions, there was no difference in the first half of trials between viewing times on the distractor letter and the solution letters (all *t*s < 1.50, n.s.). Likewise, the ANOVA revealed no significant main effects or interactions (all *F*s < 2.74, n.s.). In contrast, in the second half of trials for all conditions, a significantly greater proportion of viewing time was spent on the solution letters as compared to the distractor letter (all *t*s > 3.83, all *p*s < 0.001). In this case, the ANOVA revealed a significant main effect of letter type [*F*(1,59) = 75.65, *p* < 0.001] but no other main effects or interactions approached significance (all *F*s < 0.23, n.s.). The lack of any main effect or interaction involving central letter string type suggests that the accumulation of solution knowledge prior to insight is independent of the effects of the familiarity manipulation. Finally, we also examined the pattern of looking behavior during the first and second half of trials in which participants failed to provide a solution for the anagram. As shown in **Figures 3E,F**, failure to solve anagrams was reflected by a small but significant tendency for greater viewing time on the distractor letter relative to the solution letters [*F*(1,59) = 5.51, *p* < 0.05]. No other main effect or interaction was significant (all *F*s < 2.48, n.s.).

## DISCUSSION

The main goal of the present study was to explore the negative influence of familiarity on performance in a simplified anagram problem solving task. We replicated prior findings from the anagram literature that showed that task performance is poorer when anagrams are presented in word form than when they are presented as scrambled letters (e.g., Beilin and Horn, 1962; Ekstrand and Dominowski, 1965, 1968; Tresselt and Mayzner, 1965; Mayzner and Tresselt, 1966). This effect is thought to be due to the difficulty in breaking the gestalt of the existing word in order to rearrange the letter order and form a new word. However, participants’ eye movements in the present study revealed a more intricate pattern of the effects of stimulus familiarity on anagram problem solving, including both interference and facilitation. Specifically, the present study documented shorter viewing times on the central letter string when it was presented in word form than in nonword form, suggesting that participants were better able to encode and maintain the central letter string in working memory when it was a meaningful word than when it was a meaningless string of letters. This finding is consistent with the well established perceptual encoding and working memory advantage for familiar stimuli relative to unfamiliar stimuli (e.g., Chase and Simon, 1973; Reingold et al., 2001a).

Of more interest is what the eye movement record revealed about how familiarity interferes with anagram problem solving. Our paradigm demonstrated that the interference of stimulus familiarity was due to longer viewing times on the individual letters when the central letter string was presented as a word than when it was presented as a nonword. As originally proposed by Gestalt psychologists, these longer viewing times on the individual letters might suggest that participants find it more challenging to integrate the individual letters into the central letter string when it is a holistic entity. Supporting evidence for this possibility comes from simple letter-insertion tasks that were introduced by Reingold (1995). For example, similar to the present task, in a letter-insertion task participants were presented with a letter string that was either a word (e.g., CASH) or a nonword (e.g., CRAH) and were required to insert one of two alternative letters into the letter string to create a word (e.g., CRASH). Performance was substantially better for nonword than word letter strings (Reingold, 1995). Thus, consistent with the conceptualization of Gestalt psychology, it seems intuitive that restructuring and integrating new elements into a pre-existing holistic representation would be more difficult than integrating items into an unrelated collection of problem elements. However, more work is required in order to specify the mechanisms underlying this theoretical possibility, and to explain how familiarity interferes with the integration process.

Finally, a previous eye movement study of the Einstellung effect in chess experts suggested that the activation of familiar schemas in memory creates perceptual biases towards information that confirms these schemas and away from information that is required to find a less familiar but more optimal solution (e.g., Bilalić et al., 2008a). Our eye movement analysis did not reveal a perceptual bias towards the central letter string when it was presented in word form. This was likely due to the encoding and processing advantages that are associated with the familiar central word. These advantages might have allowed problem solvers to easily maintain familiar stimulus information in working memory while directing their visual attention elsewhere in the stimulus display. However, participants’ processing resources might have been captured by the familiar central letter string in ways that our eye movement methodology could not reveal. Specifically, it might be that the familiarity of the central letter string causes an unhelpful bias in the search for solution words based on the irrelevant orthographic, phonological, lexical, and/or semantic activation associated with the central word. Unlike previous findings which demonstrated that an exhaustive encoding mental set could be replaced by a selective encoding strategy which ignores irrelevant aspects of the stimulus regardless of stimulus familiarity (e.g., Gaschler and Frensch, 2007, 2009; see also Dreisbach and Haider, 2008, 2009), in the present study, participants were seemingly unable to ignore the irrelevant but familiar central word. This is likely due to the fact that the irrelevant activation caused by the central word was involuntary in nature and required processing resources in order to oppose it. Taken together, the present findings and prior results indicate that while stimulus familiarity and domain knowledge are clearly fundamental to establishing expertise, these aspects of skilled performance are not without their pitfalls when a problem solving scenario requires flexibility.

## Conflict of Interest Statement

The authors declare that the research was conducted in the absence of any commercial or financial relationships that could be construed as a potential conflict of interest.
